# Supplementary material for: Fast Na ion transport triggered by rapid ion exchange on local length scales
Source: Sci Rep. 2018 Aug 10;8:11970. doi: 10.1038/s41598-018-30478-7 (PMC6086902; doi:10.1038/s41598-018-30478-7)
Supplement: Supplementary file 1 — Supporting Information [file 41598_2018_30478_MOESM1_ESM.pdf]

Supporting Information

Fast Na ion transport triggered by rapid ion exchange on local length scales

S. Lunghammer,[1] D. Prutsch,[1] S. Breuer,[1] D. Rettenwander, I. Hanzu,[1,2] Q. Ma,[3] F. Tietz,[3,4] and H. M. R. Wilkening[1,2,*]

[1] *Institute for Chemistry and Technology of Materials (NAWI Graz), and Christian Doppler Laboratory for Batteries, Stremayrgasse 9, Graz University of Technology, A-8010 Graz (Austria).*

[2] *ALISTORE-ERI European Research Institute, 33 Rue Saint Leu, F-80039 Amiens (France).*

[3] *Forschungszentrum Jülich GmbH, Institute of Energy and Climate Research, Materials Synthesis and Processing (IEK-1), D-52425 Jülich (Germany)*

[4] *Helmholtz-Institute Münster, c/o Forschungszentrum Jülich GmbH, D-52425 Jülich (Germany).*

1) Conductivity isotherms (−100 … 180 ℃) and Nyquist plots

Figure **S1** shows the conductivity isotherms of Sc-NSZP highlighting the bulk and g.b. regimes (see (a)). Above 20 °C the response, if frequencies below 10^7 Hz are regarded, is only dominated by the g.b. contribution. In (b) Nyquist plots with equally scaled axis are shown. Whereas the bulk response reveals a depressed semicircle, for g.b. an almost ideal Debye relaxation is seen. In Figure **S2** (a) the corresponding conductivity isotherms of Sc-free NSZP are shown. g.b. and bulk response are of the same order of magnitude. The Nyquist plots in Figure **S2** (b) reveal two well distinguishable semicircles. See *Chem. Phys. Lett.* **701** (2018) 147 for details.

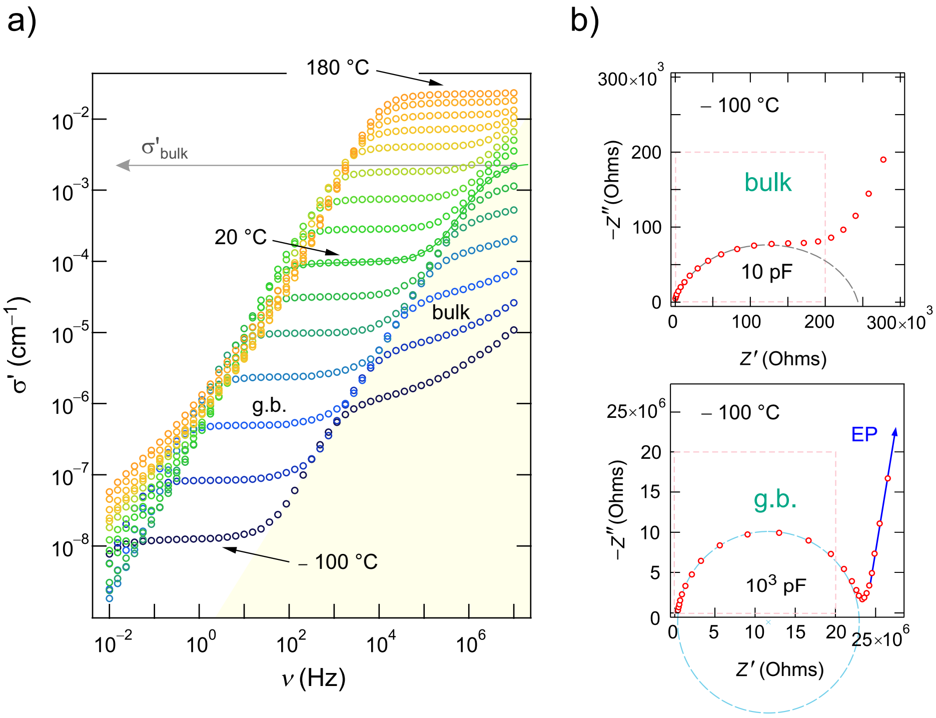

Fig. S1: a) Conductivity spectra of Sc-NSZP recorded at temperatures ranging from −100 °C up to 180 °C. The bulk plateau of the isotherm recorded at 20 °C points to a conductivity in the order of 2 mS/cm. b) Nyquist plots of the electrical response measured at −100 °C. EP means electrode polarisation.

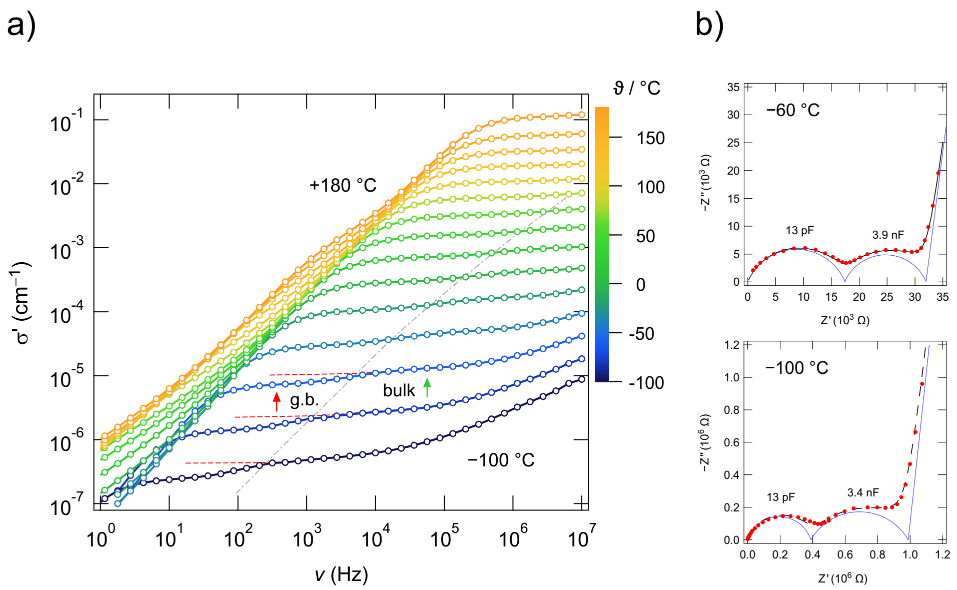

Fig. S2: a) Conductivity spectra of Sc-free NSZP. b) At temperatures well below ambient the corresponding Nyquist plots show two semicircles with capacities typical for bulk and g.b. responses.

2) ^{23}NMR (spin-lock) spin-lattice relaxation transients; NMR fitting results

Figure **S3** shows the magnetization transients $M(t)$ recorded with the spin-lock pulse sequence (a) and the saturation recovery pulse sequence (b).

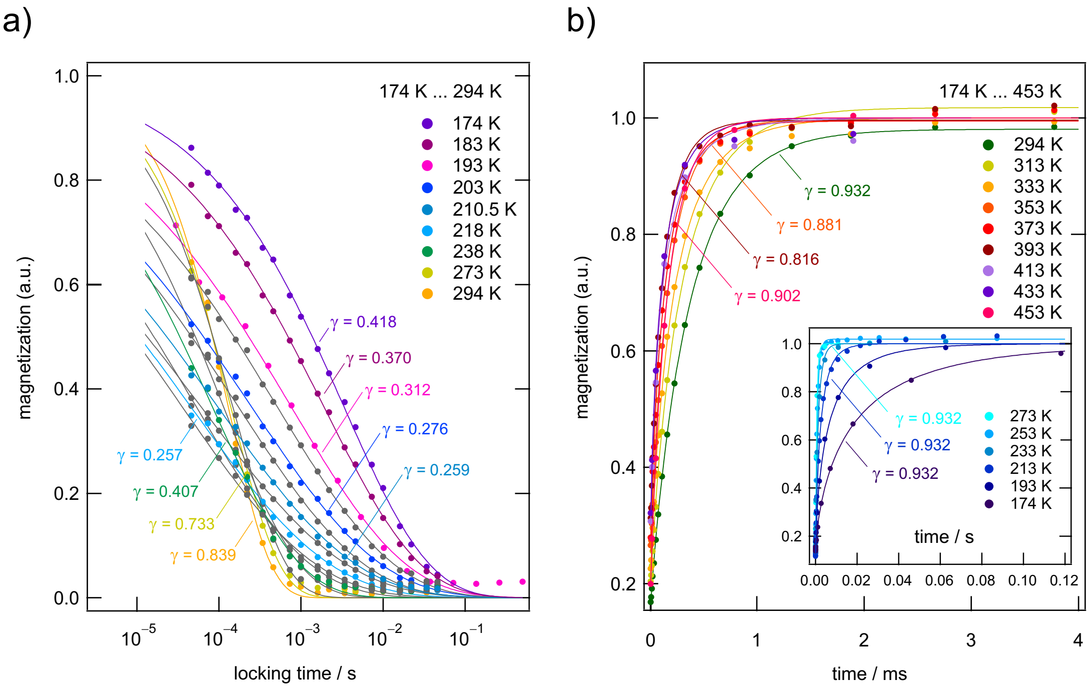

Fig. S3: a) ^{23}Na NMR spin-lock magnetization transients of Sc-NZSP used to extract the $R_{1\rho}$ rates. The solid lines show fits with stretched exponentials. γ values indicate the stretching factor that range from 0.25 to 0.84. Highly stretched exponentials are found where the rate passes through the maximum in Fig. **2c**. b) ^{23}NMR spin-lattice relaxation transients $M(t)$ obtained with the saturation recovery pulse sequence. Almost independent of temperature longitudinal magnetization recovers exponentially over the temperature range covered. The corresponding rates R_1 are shown in Fig. **2c**. The error of the rates does not exceed ±3% of the absolute value; this accuracy is needed to detect the low-T maximum in R_1 shown in Fig. **2c**.

The ^{23}Na NMR R_1 rates in Fig. **2c** were analysed with, for a good approximation, a sum of two BBP-type spectral density terms that considers deviations from 3D uncorrelated Na^+ motion. The spectral density $J(\omega_0)$ was of the form $R_1 \propto C\,[\tau_c/(1+(\omega_0\tau_c)^{\beta})]$ with τ_c being the motional correlation time, which is assumed to be in the same order of magnitude as the residence time τ between two jump processes. $\beta_{\text{(NMR)}}$ was allowed to range from 1 to 2. C denotes an overall coupling constant for the magnetic dipolar and electric quadrupolar interactions. τ_c is thermally activated with an Arrhenius law $\tau_c^{-1} = \tau_{c,0}^{-1}\exp(-E_{\text{a high-}T}/(k_B T))$. The results of our fit(s) are listed in Table **S1**.

Table S1: Results for $\tau_{c,0}^{-1}$, $E_{\text{a high-}T}$, and β_{NMR}

	$E_{\text{a high-}T}$	$\tau_{c,0}^{-1}$	β_{NMR}
peak 1, low T	0.27(1) eV	$5.8(9)\times10^{13}$ s^{-1}	1.55(2)
peak 2, higher T	0.28(3) eV	$1.2(6)\times10^{12}$ s^{-1}	1.61(2)

$\beta_{\text{NMR}} = 2$ would reflect ideal BPP behaviour, *i.e.*, uncorrelated motion, while $\beta_{\text{NMR}} < 2$ clearly indicates correlated motion. β_{NMR} is in line with α_{NMR} connecting $E_{\text{a low-}T}$ and $E_{\text{a high-}T}$. Most importantly, $\tau_{c,0}^{-1}$ values are in the order of common phonon frequencies supporting the validity of the low-T peak shown in Fig. **2c**.

3) Analysis of electrical modulus spectra

Figure **S4** presents the modulus representation of the dielectric response of Sc-NZSP. a) and b) show a log-log plot of the modulus peaks $M''(\nu)$. The peak at low frequencies refers to the g.b. response, the one shifted toward very high frequencies, see also b), corresponds to the electric response inside the grains. The characteristic frequencies read off at the peak maxima are analysed in an Arrhenius plot in the inset of b). In c) a semi-logarithmic plot of the high-frequency data is shown to better illustrate the modulus peaks.

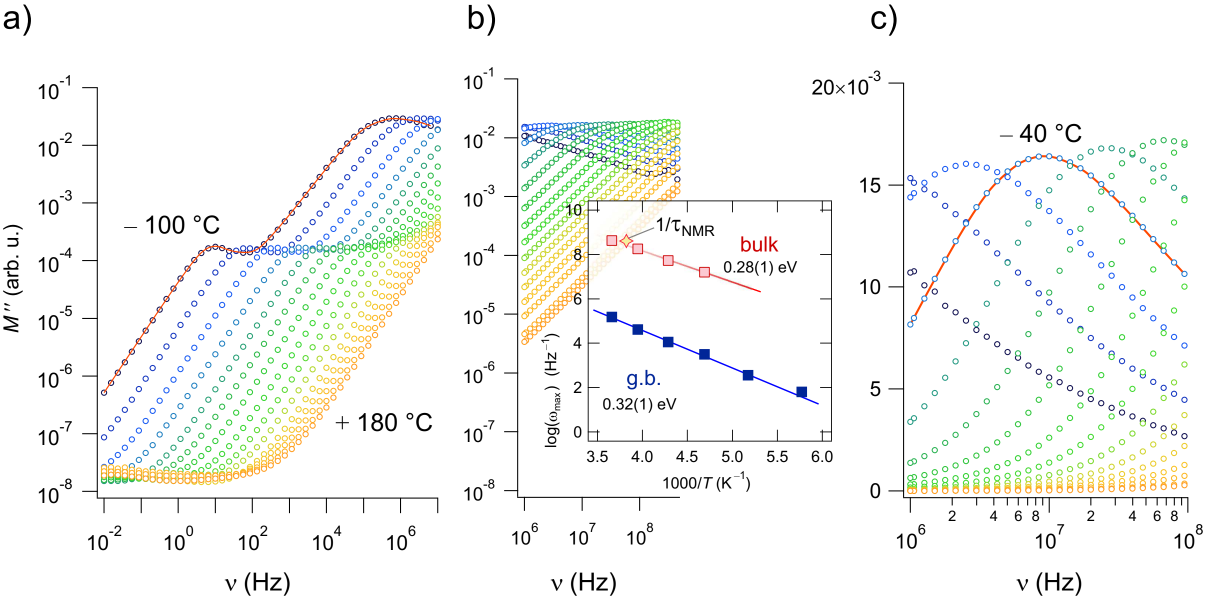

Fig. S4: a) and b) M'' representation of the electric response of Sc-NZSP. The inset shows the temperature behaviour of the relaxation frequencies for the bulk and the g.b. response. For comparison, the NMR correlation rate $1/\tau_{\text{NMR}}$ obtained from the low-T ^{23}Na $R_1(1/T)$ peak is also included. It perfectly agrees with data from broadband electric spectroscopy if carried out in the same frequency range. Note that ^{23}Na NMR was carried out at 79 MHz.
